# Differential binding of colors to objects in memory: red and yellow stick better than blue and green

**DOI:** 10.3389/fpsyg.2015.00231

**Published:** 2015-03-03

**Authors:** Christof Kuhbandner, Bernhard Spitzer, Stephanie Lichtenfeld, Reinhard Pekrun

**Affiliations:** ^1^Department of Psychology, University of Regensburg, RegensburgGermany; ^2^Department of Education and Psychology, Freie Universität Berlin, BerlinGermany; ^3^Department of Psychology, University of Munich, MunichGermany

**Keywords:** color, memory, binding, subjective confidence, red, green, blue, yellow

## Abstract

Both evolutionary considerations and recent research suggest that the color red serves as a signal indicating an object’s importance. However, until now, there is no evidence that this signaling function of red is also reflected in human memory. To examine the effect of red on memory, we conducted four experiments in which we presented objects colored in four different colors (red, green, blue, and yellow) and measured later memory for the presence of an object and for the color of an object. Across experiments, we varied the type of objects (words vs. pictures), task complexity (single objects vs. multiple objects in visual scenes), and intentionality of encoding (intentional vs. incidental learning). Memory for the presence of an object was not influenced by color. However, in all four experiments, memory for the color of an object depended on color type and was particularly high for red and yellow-colored objects and particularly low for green-colored objects, indicating that the binding of colors into object memory representations varies as a function of color type. Analyzing the observers’ confidence in their color memories revealed that color not only influenced objective memory performance but also subjective confidence. Subjective confidence judgments differentiated well between correct and incorrect color memories for red-colored objects, but poorly for green-colored objects. Our findings reveal a previously unknown color effect which may be of considerable interest for both basic color research and applied settings like eyewitness testimony in which memory for color features is relevant. Furthermore, our results indicate that feature binding in memory is not a uniform process by which any attended feature is automatically bound into unitary memory representations. Rather, memory binding seems to vary across different subtypes of features, a finding that supports recent research showing that object features are stored in memory rather independently from each other.

## INTRODUCTION

Color is a fundamental aspect of our perceptual experience of the external world and has attracted people’s interest for a long time, as can be seen in the voluminous body of research conducted over the past century to examine the physics, physiology, and psychology of color. In the domain of information processing, numerous studies have demonstrated that color is one of the basic building blocks of visual perception. For instance, it has been shown that color is an effective code to organize our visual world by grouping similar items and segregating the world into meaningful objects (Fine et al., 2003; Schulz and Sanocki, 2003).

Surprisingly, although a large amount of research has been done to determine the general role of color in cognitive processing, the question of whether specific types of color^1^ (such as red, green, blue, etc.) differentially affect perception, attention, and memory has attracted little research. However, several recent studies have increased interest in the effects of specific color categories by demonstrating that different types of color alert us to different situational requirements, based on evolutionary predispositions and learned associations (Elliot et al., 2007; Mehta and Zhu, 2009; for a review, see Elliot and Maier, 2014). One color which seems to have particular relevance is the color red. In non-human animals, red often serves as a signal that another animal or object is of importance for one’s own survival (Hutchings, 1997; Khan et al., 2011). Depending on the context, red can act as an appetitive signal (e.g., red as a signal for fertility or the ripeness of fruits; Nunn, 1999; Dominy and Lucas, 2001), or as a warning signal (e.g., red as a common aposematic color amongst terrestrial invertebrates; Edmunds, 1974). A similar link between color and stimulus importance seems to be also present in humans as can be seen, e.g., in the long-standing practice of using red lipsticks and rouge to heighten the attractiveness of women (Ragas and Kozlowski, 1998), or in the coloring of danger signals (Parsons, 1995). Indeed, recent research indicates that red can enhance the impact of external stimulation both in appetitive and aversive contexts by demonstrating that red enhances the attractiveness of women and men (Elliot and Niesta, 2008; Elliot et al., 2010), and intensifies the effect of negative stimuli (Kuhbandner and Pekrun, 2013).

The aim of the present work was to examine whether the color red also influences human memory. If red serves as a signal to indicate an object’s importance, then later memory of an object might also be enhanced when the object was colored in red. Interestingly, although several studies have shown that colored objects or scenes are generally better remembered than gray-scale images of the same items (e.g., Borges et al., 1977; Wichmann et al., 2002; Spence et al., 2006), to the best of our knowledge, it has never been reported that red-colored objects are more likely to be remembered than other-colored objects. This may simply reflect a gap in prior research. However, it might also be that red does not influence whether information about the presence or absence of an object is retained, but rather whether information about the color of an object is stored in memory. Indeed, such a hypothesis can also be derived from an evolutionary perspective. As outlined above, red seems to serve the distinction between different exemplars of the same type according to, e.g., their state of ripeness or fertility, rather than signaling the general presence of types of exemplars. Accordingly, red may not have any specific function for remembering that there was an exemplar per se, but may produce an enhanced binding of colors to exemplars in memory. Such an enhanced binding may be highly adaptive to memorize the significance of individual objects for a person’s issues and goals.

In fact, such a prediction can also be derived from previous findings on the effect of colors on cognitive processes that are assumed to underlie the binding of features into object representations. As elaborated in the Feature-integration theory (Treisman and Gelade, 1980), the different features of an object (e.g., color, shape, orientation, etc.) are first registered automatically and parallel in independent feature stores, and in order to bind the features into object representations, attention is required. Evidence for this notion comes from studies showing that when attention is diverted, features of presented objects are sometimes erroneously recombined, producing illusory conjunctions (e.g., Treisman and Schmidt, 1982). Thus, given that recent research has shown that the features of objects are stored in visual memory independently from each other rather than within a single unitary representation (Fougnie and Alvarez, 2011; Brady et al., 2013), the binding of features should vary as a function of how strongly different types of features attract attention. With regard to color, the color that most strongly attracts attention seems to be red. Such an assumption has initially been derived from studies in applied contexts such as advertising, showing that red is the most arousing, exciting, and stimulating color (e.g., Labrecque and Milne, 2011; see Fraser and Banks, 2004, for a review), and has been supported by more basic research showing that red is more salient than other colors (e.g., Gelasca et al., 2005; Frey et al., 2011). Accordingly, color features may attract more attention when being red, leading to an enhanced binding of red colors into object memory representations.

**FIGURE 1:**
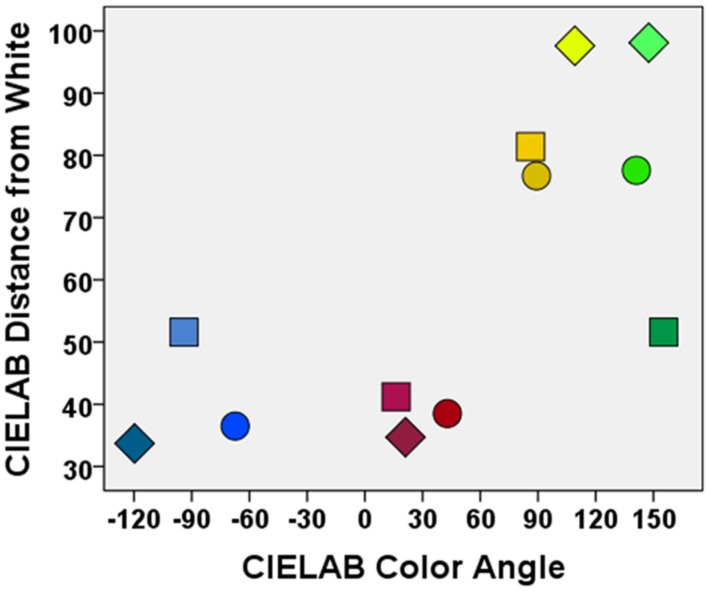
**Illustration of the colors used in the four experiments.** The colors of the symbols show the colors of the stimuli in Experiment 1 (squares), Experiment 2 (disks), and Experiments 3 and 4 (diamonds). In Experiment 1, focal colors were used to control for possible effects of color typicality. In Experiment 2, colors were pairwise equated on lightness, and in Experiments 3 and 4 additionally on saturation, to account for possible confounding effects of these color attributes. Note that colors will not be correctly displayed in print or on an uncalibrated video monitor.

**FIGURE 2:**
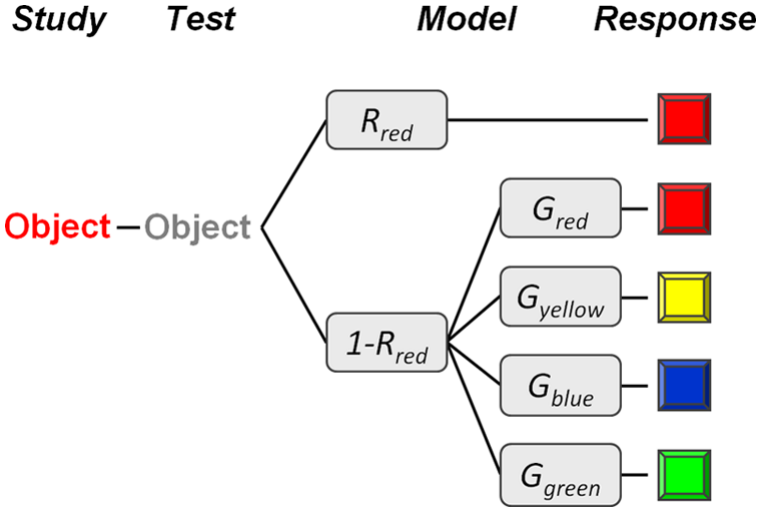
**Illustration of the multinomial model.** Subjects are assumed to have a veridical memory of an object’s original color (e.g., red) with probability *R* (e.g., *R_red_*), and to guess one of the four colors with the probability *G* (e.g., *G_red_*) in the absence of memory for the object’s original color.

To examine whether the binding of colors to objects in memory varies as a function of color type, we conducted four experiments. In all of the experiments, we investigated the effects of coloring objects in four different basic colors (red, blue, yellow, and green; see **Figure 1**) on later memory for the presence of an object and for the color of an object. The colors red, blue, yellow, and green were chosen because they represent psychological primary colors and belong to the limited number of basic colors which can be internally represented and uniquely identified across different cultures (Hård and Sivik, 1981; Regier et al., 2005). In all four experiments, none of the used objects were pre-experimentally associated with any particular color. As long-term memory of colors is characterized by color categories rather than by the exact colorimetric properties of the originally perceived color stimuli (e.g., Heider, 1972; Uchikawa and Shinoda, 1996; Regier et al., 2005), we asked participants in the memory test to provide a categorical memory response by making a forced-choice among the four possible color types. To account for possible effects of biased responding or guessing (e.g., a general tendency to respond “red”), we applied a simple multinomial model to the data of each experiment (see **Figure 2**), allowing us to quantify color-specific memory separately from color-specific guessing. To further assess whether color not only influences objective memory performance but also subjective confidence in memories, in all experiments, participants additionally rated their confidence in their color memories (from 1 = extremely uncertain to 5 = extremely certain). Across the four experiments, we varied (i) the type of objects (words vs. pictures), (ii) task complexity (single objects vs. multiple objects in visual scenes), and (iii) intentionality of encoding (intentional vs. incidental learning).

## EXPERIMENT 1: VERBAL MEMORY

**FIGURE 3:**
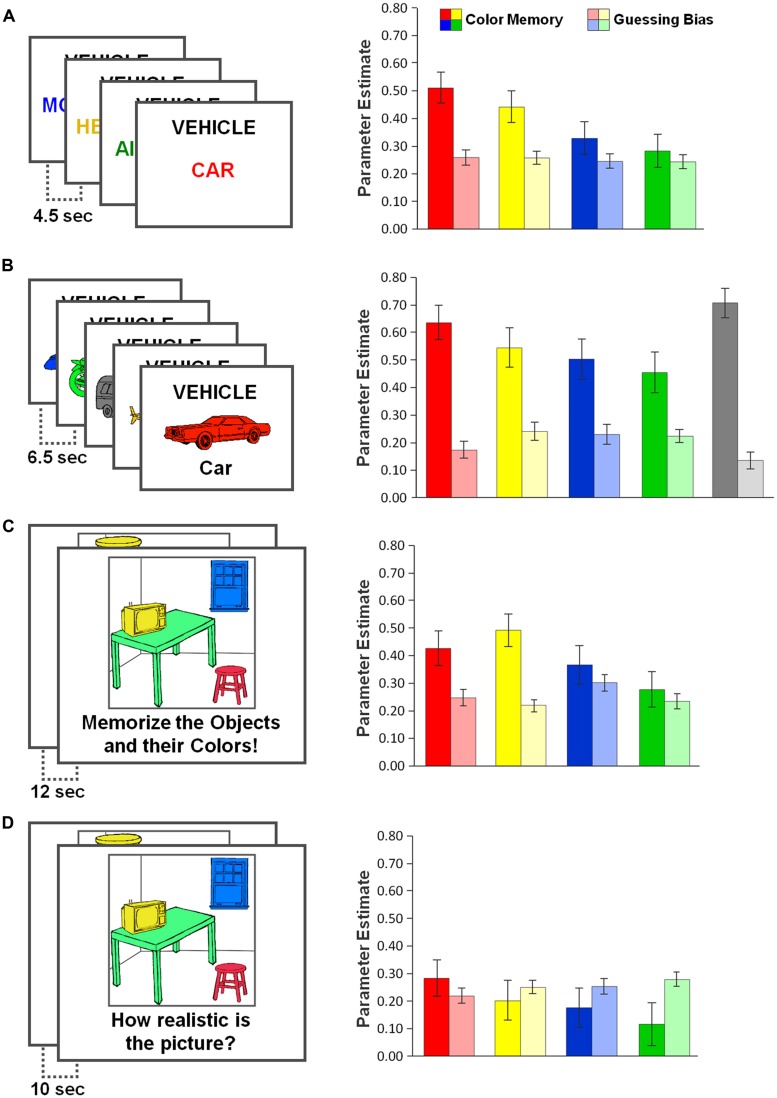
**Study materials (left panels) and results (right panels) of the four experiments: **(A)** words, **(B)** pictures,** (C)** visual scenes, and** (D)** incidental learning.** Saturated colors indicate parameter estimates for color-specific memory (i.e., probability of remembering an object’s color), pale colors indicate parameter estimates for color-specific guessing (i.e., the tendency to respond with a specific color in the absence of memory for an object’s original color). Parameters were estimated using the multinomial model shown in **Figure 2**. Error bars represent 95% confidence intervals of the parameter estimates.

In Experiment 1, we examined the effect of specific colors on verbal learning. Participants were presented names of prototypic exemplars of semantic categories one by one in red, blue, yellow, or green font (see **Figure 3A**, left panel), with the instruction to memorize each exemplar as well as each exemplar’s color for a later memory test. As previous research has shown that primary color categories are organized around universally shared focal points in color space (e.g., Boynton et al., 1989; Regier et al., 2005), we used focal colors as the best examples of a color category in Experiment 1 (for an illustration of the colors used in the experiments, see **Figure 1**).

### MATERIALS AND METHODS

#### Participants

Forty-eight (31 females, *M* age = 24.3 years) undergraduate students participated in the experiment for course credit. They were tested individually. Participation was restricted to individuals who were not colorblind (based on self-report). All experiments were approved according to the ethical standards at the University of Munich where the experiments were conducted.

#### Materials

The study list consisted of six semantic categories (fish, furniture, musical instruments, occupations, tools, vehicles) which contained eight exemplars each (e.g., vehicles – car; taken from Mannhaupt, 1983). The initial letter of each word was unique within its category. Two of the eight exemplars of a category were presented in red, two in green, two in blue, and two in yellow font. The assignment of colors to exemplars was counterbalanced across participants. In all experiments, colors were chosen using a spectrometer (i1Pro, X-rite Inc., Grandville, MI, USA) according to the device independent CIELAB color space (Wyszecki and Stiles, 1982). CIELAB coordinates are always specified with respect to a reference white point, which was in our study a white light metameric to 60 cd/m2 CIE Standard Illuminant C [L^∗^a^∗^b^∗^(100.0, 0.0, 0.0)]. In Experiment 1, focal colors were used to control for possible effects of color typicality [colors were chosen according to Regier et al., 2005; *red*: L^∗^a^∗^b^∗^(41.2, 61.4, 17.9), *blue*: L^∗^a^∗^b^∗^(51.6, -3.4, -48.1), *yellow*: L^∗^a^∗^b^∗^(81.4, 7.3, 109.12), *green*: L^∗^a^∗^b^∗^(51.6, -63.3, 29.0)].

#### Procedure

In the study phase, the exemplar names were presented together with their category names one by one in random order for 4 s each with an interstimulus interval of 0.5 s. In all four experiments, the stimuli were presented on a white background using Eprime software version 2.0 (Psychology Software Tools, Inc., Pittsburgh, PA, USA). The order of presentation was determined by blocked randomization. A random sequence of eight blocks was presented consisting of one randomly selected exemplar from each of the six categories. Participants were instructed to memorize each exemplar as well as each exemplar’s color for a later memory test. The study procedure was identically repeated with another random serial order of the study items. After a 4-min distractor task (solving simple arithmetic problems), memory for exemplars was tested first by presenting the first letter of each studied exemplar together with its category name for 4.5 s. This was done to control for possible effects of output interference. Order of presentation was blocked by category; within a category, the presentation of the first letters was randomized. The memory test for the exemplars’ colors followed. Each studied exemplar was presented in gray font together with its category name, and participants were instructed to indicate in which color it was presented in the study phase by pressing one of four different buttons showing a red, blue, yellow, or green color. Each button was equally often provided with a specific color across all participants. Presentation order was again blocked by category. If the participants did not remember the color of an exemplar, they were instructed to guess. In all four experiments, after each color response, participants were asked to rate their confidence in their color response on a 5-point rating scale ranging from 1 = extremely uncertain to 5 = extremely certain.

### RESULTS

**Table 1** gives an overview of the probabilities observed in the four experiments for (1) correctly remembering the presence of an object, (2) correctly classifying the color of an object, and (3) falsely reporting a specific color when misclassifying the color of an object.

**Table 1:** Overview of the memory results of the four experiments.

	Probability of recalling the presence of an object				Probability of correctly classifying an object’s color				Probability of misclassifying a color			
*Experiment*	Red	Yellow	Blue	Green	Red	Yellow	Blue	Green	Red	Yellow	Blue	Green
*Verbal memory*	0.53 (0.18)	0.55 (0.20)	0.51 (0.17)	0.49 (0.17)	0.64 (0.21)	0.59 (0.23)	0.50 (0.24)	0.45 (0.21)	0.34 (0.17)	0.34 (0.17)	0.33 (0.15)	0.30 (0.17)
*Visual memory*	0.49 (0.17)	0.47 (0.20)	0.47 (0.17)	0.40 (0.18)	0.70 (0.25)	0.69 (0.25)	0.62 (0.27)	0.58 (0.29)	0.22 (0.17)	0.33 (0.23)	0.27 (0.15)	0.27 (0.16)
*Visual scenes*	0.57 (0.23)	0.60 (0.23)	0.56 (0.22)	0.45 (0.26)	0.55 (0.28)	0.62 (0.27)	0.53 (0.28)	0.46 (0.29)	0.33 (0.14)	0.28 (0.15)	0.38 (0.20)	0.34 (0.19)
*Incidental learning*	0.51 (0.20)	0.44 (0.19)	0.49 (0.18)	0.49 (0.15)	0.46 (0.18)	0.39 (0.16)	0.40 (0.24)	0.33 (0.20)	0.30 (0.17)	0.34 (0.12)	0.35 (0.12)	0.34 (0.17)

*SD are given in parentheses. In the experiment on visual memory, there was an additional color condition (gray: *M*_Object_ = 0.43, SD = 0.20; *M*_Color_ = 0.72, SD = 0.20; *M*_Misclassification_ = 0.15, SD = 0.13).*

In Experiment 1, the probability of correctly remembering the presence of an object was not influenced by color [*F*(3,141) = 1.41, *MSE* = 0.020, *p* = 0.244, $\eta_{p}^{2}$ = 0.03; mean (*M*) recall = 0.52]. However, the probability of correctly classifying the color of an exemplar depended on type of color [*F*(3,141) = 13.16, *MSE* = 0.025, *p* < 0.001 $\eta_{p}^{2}$ = 0.22], and was high for red and yellow-colored exemplars (*M* red = 0.64; *M* yellow = 0.59), compared to blue and green-colored exemplars (*M* blue = 0.50; *M* green = 0.45). By contrast, when the color of an exemplar was misclassified, the probability of erroneously reporting one of the remaining colors did not differ across color types [*F*(3,141) = 0.36, *MSE* = 0.036, *p* = 0.782, $\eta_{p}^{2}$ < 0.01].

In order to separately quantify color-specific memory and color-specific guessing, we applied a simple multinomial model to the data (see **Figure 2**). Multinomial models describe categorical response probabilities as a function of discrete cognitive states and can be represented as hierarchical process tree structures (Batchelder and Riefer, 1999). In our model, we assume that subjects correctly remember an item’s original color (e.g., red) with probability *R* (e.g., *R_red_*). In the absence of memory for the item’s original color, with a probability of 1-*R_red_*, subjects are assumed to guess one of the four colors. Color-specific guessing (i.e., guessing bias) was modeled by a multinomial parameter set *G*, with the restriction *G_red_ = 1–G_blue_–G_yellow_–G_green_*. When applied jointly to the response distributions for each of the four item types, the model had seven free parameters (*R_red_*, *R_blue_*, *R_yellow_*, *R_green_*, *G_blue_*, *G_yellow_*, *G_green_*) to fit 12 independent data points (3 independent responses × 4 object colors). Thus, the model had 5^∘^ of freedom for testing its goodness of fit. The model parameters were estimated using maximum-likelihood techniques, which also allow for statistical testing.

The multinomial model described the data well in Experiment 1 [χ^2^(5) = 2.54, *p* = 0.771]. The multinomial-model based parameter estimates for color-specific memory (*R*) and color-specific guessing (*G*) in Experiment 1 are shown in **Figure 3A** (right panel). Replicating the above ANOVA results, Likelihood-ratio tests revealed significant variations in the memory parameter *R* [χ^2^(3) = 34.54, *p* < 0.001], but not in the guessing parameter *G* [χ^2^(2) < 1]. Planned pairwise comparisons confirmed that parameter *R* was significantly increased for red exemplars compared to blue [χ^2^(1) = 18.02, *p* < 0.001] and green exemplars [χ^2^(1) = 27.76, *p* < 0.001], and there was a slight trend for *R* being higher for red compared to yellow exemplars [χ^2^(1) = 2.71, *p* = 0.100].

### DISCUSSION

The results of Experiment 1 demonstrate that the probability of remembering the color in which a word was presented during initial study varies substantially as a function of color type. In line with the assumption that red may bring about an enhanced binding of color features because red often serves as a signal that a stimulus is of importance for one’s own survival (Hutchings, 1997; Khan et al., 2011), memory for the color of studied words was higher for red than for blue and green-colored words. Such a finding indicates for the first time that feature binding in memory is not a uniform process by which any attended feature of a stimulus is automatically bound into memory representations. Rather, our results suggest that particularly important features are more strongly bound, possible due to increased attentional attraction during initial encoding (e.g., Treisman and Gelade, 1980), a finding that supports recent findings showing that the features of objects are stored in visual memory rather independently from each other (Fougnie and Alvarez, 2011; Brady et al., 2013).

Although memory for the color of red-colored words was descriptively higher than for yellow-colored words, the difference failed to reach significance. This may simply reflect the fact that the power of Experiment 1 was too small to detect small-sized effects. However, given that both red and yellow are linked to aposematism in insects and reptiles (e.g., Stevens and Ruxton, 2012) and commonly used to indicate caution in signage and brake lights in human culture (Parsons, 1995), it may also indicate that binding is also increased for yellow colors. In order to replicate the findings of Experiment 1 and to further explore the role of color type in memory binding, we conducted a second Experiment in which we examined the binding of color features in visual memory.

## EXPERIMENT 2: VISUAL MEMORY

The aim of Experiment 2 was to replicate the findings of Experiment 1 with visual stimulus materials, and to explore the possible role of attentional attraction. The design and procedure were similar to Experiment 1 with the main difference that pictures of exemplars instead of names were used as stimuli that were filled with either red, blue, yellow, or green colors (see **Figure 3B**, left panel). To account for possible confounding effects of low-level color attributes in Experiment 1 where focal colors were used that represent the best examples of a color category but vary in lightness, in Experiment 2 the colors were pairwise equated on lightness (i.e., red/blue and yellow/green). We expected to find a similar pattern of results than that observed in Experiment 1, with better color memory when visual objects were initially colored red than when they were colored yellow, blue or green.

Furthermore, in order to investigate whether the enhanced binding for red color may be explained by the assumption that increased attention to a color feature can produce an enhanced binding of that feature into object memory representations (e.g., Treisman and Gelade, 1980; Wheeler and Treisman, 2002), we additionally included oddball color pictures (i.e., achromatic pictures that occurred only rarely within the sequence of mostly chromatic pictures). As oddball stimuli are known to attract attention (Remington et al., 1992), memory for the color of oddball pictures should also be enhanced, even although achromatic colors seem not to serve any signaling function (e.g., Parsons, 1995; Stevens and Ruxton, 2012).

### MATERIALS AND METHODS

#### Participants

Forty (31 females, *M* age = 24.0 years) undergraduate students participated in the experiment for course credit. They were tested individually. Participation was restricted to individuals who were not colorblind (based on self-report).

#### Materials

The material was similar to that used in Experiment 1 with the only difference that pictures instead of names of exemplars were used as stimuli. The study list consisted of four categories which contained drawings of 10 prototypic exemplars (taken from Snodgrass and Vanderwart, 1980). Two were colored in red, two in green, two in blue, and two in yellow, the remaining two exemplars showed an achromatic color (gray). To examine whether the effects of different colors on binding observed in Experiment 1 depend on specific low-level physical color attributes, and to additionally account for possible confounding effects of variations in lightness, we slightly varied the colors used in Experiment 1 and equated the colors pairwise on lightness (i.e., for red-blue, and for green-yellow). Colors were pairwise equated because equating all four colors on lightness would have resulted in relatively untypical colors. Equated here means functionally equivalent [i.e., within two units on the relevant parameter L; see ref. 33; *red*: ^∗^La^∗^b^∗^(38.5, 69.7, 64.7), *blue*: L^∗^a^∗^b^∗^(36.5, 45.5, -109.4), *yellow*: L^∗^a^∗^b^∗^(76.7, 1.1, 79.4), *green*: L^∗^a^∗^b^∗^(77.6, -85.8, 69.2), *gray*: L^∗^a^∗^b^∗^(37.0, 0.0, 0.0)]. The assignment of colors to exemplars was counterbalanced across participants.

#### Procedure

The procedure was the same as in Experiment 1. In the study phase, the drawings of the exemplars were presented in the middle of the screen one by one in random order together with their category name and the exemplar name (to standardize later retrieval) for 6 s each. Presentation time was slightly increased because of the higher complexity of stimuli. After a 5-min distractor task (solving simple arithmetic problems), memory for exemplars was tested first by presenting the first letter of an exemplar together with its category name. Order of presentation was blocked by category; within a category, the presentation of the first letters was randomized. The memory test for the exemplars’ colors followed. Each exemplar drawing was presented in black-and-white, and participants were asked to indicate in which color it was initially presented by pressing one of five different buttons showing a red, blue, yellow, green, or gray color.

### RESULTS

Memory test performance for the presence of an exemplar and the color of an exemplar are shown in **Table 1**. Memory for the presence of an exemplar was not influenced by color [*F*(4,156) = 1.92, *MSE* = 0.026, *p* = 0.110, $\eta_{p}^{2}$ = 0.047; *M* recall = 0.45]. However, replicating the results from Experiment 1, the probability of correctly classifying the color of an exemplar depended on type of color [*F*(4,156) = 4.05, *MSE* = 0.038, *p* = 0.004, $\eta_{p}^{2}$ = 0.094], and was high for red, yellow, and gray-colored exemplars (*M* red = 0.70; *M* yellow = 0.69; *M* gray = 0.72), compared to blue and green-colored exemplars (*M* blue = 0.62; *M* green = 0.58). The probability of erroneously reporting one of the remaining colors when the color of an exemplar was misclassified did also vary with color type [*F*(4,148) = 4.87, *MSE* = 0.037, *p* = 0.001, $\eta_{p}^{2}$ < 0.116]; however, this effects was mainly driven by the low misclassification rate observed for gray colored exemplars (*M*_false_ gray = 0.15), whereas the misclassification rate did not differ between the other four colors [*F*(3,111) = 2.10, *MSE* = 0.041, *p* = 0.104, $\eta_{p}^{2}$ = 0.054; *M*_false_ red = 0.22, *M*_false_ yellow = 0.33, *M*_false_ blue = 0.27, *M*_false_ green = 0.27).

The multinomial model did not optimally fit the data [χ^2^(11) = 21.34, *p* = 0.030]. Still, stable maximum likelihood estimates could be derived for each of the model’s parameters. Replicating the above ANOVA results, likelihood-ratio tests (**Figure 3B**, right panel) revealed significant variations in the memory parameter *R* [χ^2^(4) = 30.86 *p* < 0.001], and also in the guessing parameter *G* [χ^2^(3) = 25.23, *p* < 0.001]. Planned pairwise comparisons confirmed that for red-colored exemplars, parameter *R* was significantly increased compared to blue-colored exemplars [χ^2^(1) = 7.62, *p* = 0.007] and green-colored exemplars [χ^2^(1) = 13.13, *p* < 0.001], but was not statistically different from yellow [χ^2^(1) < 1] or gray-colored exemplars [χ^2^(1) = 1.26, *p* = 0.262]. With respect to parameter *G*, the data suggest that gray, but also red, were guessed relatively rarely (see **Figure 3B**).

### DISCUSSION

The results of Experiment 2 closely replicate those of Experiment 1. The probability of remembering the color in which an object was presented during initial study varied as a function of color type, with memory for the color of objects being higher for red than for blue and green-colored objects. Thus, an enhanced binding of red colors seems to be a rather fundamental phenomenon that is found both in verbal and visual memory. The results for the gray-colored oddball objects suggest that an increased binding of features can indeed be brought about by attentional attraction. Although gray color seems not to serve any signaling function (e.g., Parsons, 1995; Stevens and Ruxton, 2012), memory for the color of oddball objects was as high as memory for the color of red-colored objects. As oddball stimuli attract attention (Remington et al., 1992), such a finding supports the assumption that increased attention to a color feature can produce an enhanced binding of that feature (e.g., Treisman and Gelade, 1980; Wheeler and Treisman, 2002), a mechanism that may also underlie the effect of red on binding.

As in Experiment 1, although memory for the color of red-colored objects was descriptively slightly higher than for yellow-colored objects, the difference failed to reach significance. Given that no statistically significant difference was again observed between red and yellow, this seems to reflect the fact that red and yellow do not differ with respect to binding strength, rather than the problem of too low power to detect small effect sizes. In order to further replicate the findings of Experiment 1 and 2, and to examine whether the observed effects generalizes to visual scenes consisting of several differentially colored objects, a third experiment was conducted.

## EXPERIMENT 3: VISUAL SCENES

In Experiments 1 and 2, the to-be-studied stimuli were presented one by one. The aim of Experiment 3 was to examine whether a differential binding of colors to objects occurs also when differentially colored objects are embedded in visual scenes so that all colors are present during a study trial. The material, design, and procedure were similar to Experiment 2 with the difference that four different objects colored in red, blue, yellow, or green were together shown in simple visual scenes (see **Figure 3C**, left panel). In addition, to further address the role of potentially confounding effects of low-level color attributes, in Experiment 3, the used colors were additionally pairwise equated on saturation. We expected to find a similar pattern of results than that observed in Experiments 1 and 2, with better color memory when visual objects were colored red than when they were colored yellow, blue, or green.

### MATERIALS AND METHODS

#### Participants

Forty-eight (31 females, *M* age = 28.0 years) undergraduate students participated in the experiment for course credit. They were tested individually. Participation was restricted to individuals who were not colorblind (based on self-report).

#### Materials

The material was the same as that used in Experiment 2 with the only difference that the drawings of the exemplars were not presented one by one, but embedded in simple visual scenes. Ten visual scenes were created containing four different objects each, colored in red, blue, yellow, or green. The assignment of colors to objects was counterbalanced across participants. To additionally account for possible confounding effects of variations saturation, the colors were pairwise equated on both lightness and saturation [i.e., for red-blue, and for green-yellow; red: L^∗^a^∗^b^∗^ (34.7, 50.8, 19.6), blue: L^∗^a^∗^b^∗^ (33.7, -27.4, -48.0), yellow: L^∗^a^∗^b^∗^ (97.6, -34.9, 100.3), and green: L^∗^a^∗^b^∗^ (98.1, -90.1, 57.2)].

#### Procedure

The procedure was similar to that used in Experiments 1 and 2. In the study phase, the visual scenes were presented one by one in random order for 12 s each with the instruction to memorize the objects as well as their colors shown in the visual scenes for a later memory test. After a 2-min distractor task (solving simple arithmetic problems), a free-recall test followed in which participants were instructed to write down as many of the previously presented objects as possible for 2 min. A free recall test was chosen because the study material was not presented in a categorized way during study. A test for the memory for the objects’ colors followed in which each object was presented in black-and-white and participants were asked to indicate in which color it was initially presented by pressing one of four different buttons showing a red, blue, yellow, or green color.

### RESULTS

Memory for the presence of an object and the color of an object are shown in **Table 1**. Probability of recalling an object varied as a function of color in which the object was presented during study [*F*(3,141) = 9.68, *MSE* = 0.023, *p* < 0.001, $\eta_{p}^{2}$ = 0.171]. Green-colored objects were remembered worst (*M* green = 0.45), compared to objects colored in one of the other three colors (*M* red = 0.57, *M* blue = 0.56, *M* yellow = 0.60; all *t*s > 3.53, all *p*s < 0.001), which did not differ from each other [*F*(2,94) = 1.15, *MSE* = 0.024, *p* = 0.321]. The probability of correctly classifying the color of an object also depended on type of color [*F*(3,141) = 4.69, *p* = 0.004, $\eta_{p}^{2}$ = 0.091], and was higher for red, blue, and yellow-colored objects (*M* red = 0.55; *M* blue = 0.53; *M* yellow = 0.62), compared to green-colored objects (*M* green = 0.46). The probability of erroneously reporting one of the remaining colors when the color of an object was misclassified did not significantly vary with color type [*F*(3,141) = 1.89, *MSE* = 0.039, *p* = 0.134, $\eta_{p}^{2}$ = 0.039].

The multinomial model described the data well [χ^2^(5) = 7.36, *p* = 0.195]. The results (**Figure 3C**, right panel) showed significant color-specific differences in the memory parameter *R* [χ^2^(3) = 23.55, *p* < 0.001], and also in the guessing parameter *G* [χ^2^(2) = 10.69, *p* = 0.014]. Planned pairwise comparisons showed that parameter *R* was of comparable size for red items compared to yellow [χ^2^(1) = 2.17, *p* = 0.141] and blue items [χ^2^(1) = 1.45, *p* = 0.229], but was significantly higher for red compared to green items [χ^2^(1) = 9.72, *p* = 0.002]. With respect to color-specific guessing, *post hoc* analysis showed that the probability of guessing “blue” was significantly higher than random unbiased guessing [0.30 vs. 0.25, χ^2^(1) = 10.08, *p* = 0.001]^2^.

### DISCUSSION

Replicating the results of Experiments 1 and 2, the probability of remembering the color in which an object was visually presented during initial study varied as a function of color type, with memory for the color of an object being higher for red than for green-colored objects. Furthermore, again no statistically significant difference was observed between red and yellow, further supporting the view that red and yellow do not differ with respect to binding strength. Other than in Experiments 1 and 2, although memory for the color of an object was descriptively higher for red compared to blue-colored objects, the difference failed to reach significance. However, this may simply be a matter of power because even when an effect is true, some experiments will generate samples that do not satisfy the criterion for statistical significance (e.g., Ioannidis and Trikalinos, 2007). In order to further explore the role of color type in the binding of color features, we conducted a fourth experiment in which we tried to replicate the findings of Experiment 3 under incidental learning conditions.

## EXPERIMENT 4: INCIDENTAL LEARNING

One characteristic of Experiments 1 to 3 is that participants were instructed to study both the exemplars and the colors of the exemplars for a later memory test in the study phase. Thus, it may be that the enhanced binding of red colors observed in Experiments 1 to 3 may reflect an effect that is based on more strategic components of memory binding such as differential rehearsal strategies (e.g., Cuvo, 1975). To rule out any effects of encoding strategies in Experiment 4, we examined whether the binding of colors into memory representations varies even as a function of color type when objects are processed without any intention of memorization (i.e., incidental learning). The design and procedure of Experiment 4 were similar to Experiment 3 with the only difference that participants were not instructed to memorize the visual scenes for a later memory test. Instead, they were presented the visual scenes with the instruction to judge how realistic each pictures was (see **Figure 3D**, left panel). A surprise memory test followed in which memory for the presence of objects and the objects’ colors were tested.

Previous research has demonstrated that observers show substantial memory for the color of perceived objects even when objects were presented under incidental learning conditions (e.g., Brady et al., 2013), indicating that long-term memory representations of perceived objects are incidentally formed as a natural product of perception. Thus, finding an increased memory for red colors even when observers do not have any intention of remembering the perceived objects later would indicate that the enhanced binding of red automatically occurs as a basic phenomenon of our processing of the external world.

### MATERIALS AND METHODS

#### Participants

Forty-eight (31 females, *M* age = 25.2 years) undergraduate students participated in the experiment for course credit. They were tested individually. Participation was restricted to individuals who were not colorblind (based on self-report). None of the participants expected that they would be tested on memory later as indicated by a post-experimental questionnaire.

#### Materials and procedure

The material was the same as that used in Experiment 3. The procedure was also similar to Experiment 3 with the only exception that participants were not told to memorize the visual scenes, but instructed to judge how realistic each pictures was. No mention was made that memory would be tested later. Eight visual scenes containing four different objects colored in red, blue, yellow, or green were presented one by one in random order for 10 s each. The assignment of colors to objects was counterbalanced across participants. After the presentation of a scene, participants were instructed to evaluate how realistic the scene was using a five-point rating scale (from 1 = unrealistic to 5 = very realistic). After a 2-min distractor task (solving simple arithmetic problems), a surprise memory test followed in which first a free-recall test on memory for the presence of objects was conducted, followed by a memory test for the objects’ colors (for details, see Experiment 3).

### RESULTS

Memory for the presence of an object and the color of an object are shown in **Table 1**. Memory for the presence of an object was not influenced by color [*F*(3,141) = 1.40, *MSE* = 0.025, *p* = 0.245, $\eta_{p}^{2}$ = 0.029; *M* recall = 48.1%]. However, the probability of correctly classifying the color of an object depended on color type [*F*(3,141) = 4.69, *p* = 0.004, $\eta_{p}^{2}$ = 0.091] and was high for red-colored objects (*M* red = 0.46), medium for blue and yellow-colored objects (*M* blue = 0.40; *M* yellow = 0.39), and low for green-colored objects (*M* green = 0.33). The probability of erroneously reporting one of the remaining colors when the color of an exemplar was misclassified did not significantly vary with color type [*F*(3,141) = 0.36, *MSE* = 0.036, *p* = 0.782, $\eta_{p}^{2}$ = 0.008].

The multinomial model did not optimally fit the data [χ^2^(5) = 12.89, *p* = 0.024]. Still, stable maximum likelihood estimates could be derived for each of the model’s parameters. Replicating the above ANOVA results, the multinomial model results (**Figure 3D**, right panel) showed significant variations in the memory parameter *R* [χ^2^(3) = 10.10, *p* = 0.018, but not in the guessing parameter *G* [χ^2^(2) = 2.72, *p* = 0.257]. Planned pairwise comparisons confirmed that parameter *R* was significantly increased for red objects compared to blue [χ^2^(1) = 4.41, *p* = 0.036] and green objects [χ^2^(1) = 9.52, *p* = 0.002], but was not different compared to yellow objects [χ^2^(1) = 2.49, *p* = 0.115].

### DISCUSSION

The results of Experiment 4 closely replicate the pattern of results observed in Experiments 1 to 3. The probability of remembering the color in which an object was presented during initial study varied as a function of color type. Memory for the color of objects was higher for red than for blue and green-colored objects, and again, although memory for red colors was descriptively higher than for yellow colors, no statistically significant difference was observed between red and yellow colors. These findings demonstrate that the binding of color features into object memory representations varies as a function of color type even when observers do not have any intention of remembering the perceived objects later. Thus, the enhanced binding of red colors in memory seems to be a natural product of perception.

## COMBINED DATA SET

The pattern of results observed across the four Experiments for the memory of the color of an object was rather similar. Memory for the color of an object was higher for red-colored objects compared to blue and green-colored objects, whereas no significant difference in color memory between red and yellow-colored objects was observed. However, statistically, the difference between red and blue-colored objects failed to reach significance in one Experiment (visual scenes), and even though there was no statistically significant difference observed between red and yellow-colored objects, descriptively, memory for red colors was slightly higher than for yellow colors in all but one of the experiments (visual scenes). Both aspects may reflect matters of power because when power is not extremely high, true effects may not necessarily always be reflected in statistically significant effects, especially when effect sizes are small (e.g., Ioannidis and Trikalinos, 2007). Accordingly, in order to increase power and get a more robust estimate of the effects of colors, we finally combined the data from the four experiments.

### MEMORY PERFORMANCE FOR COLORS

**FIGURE 4:**
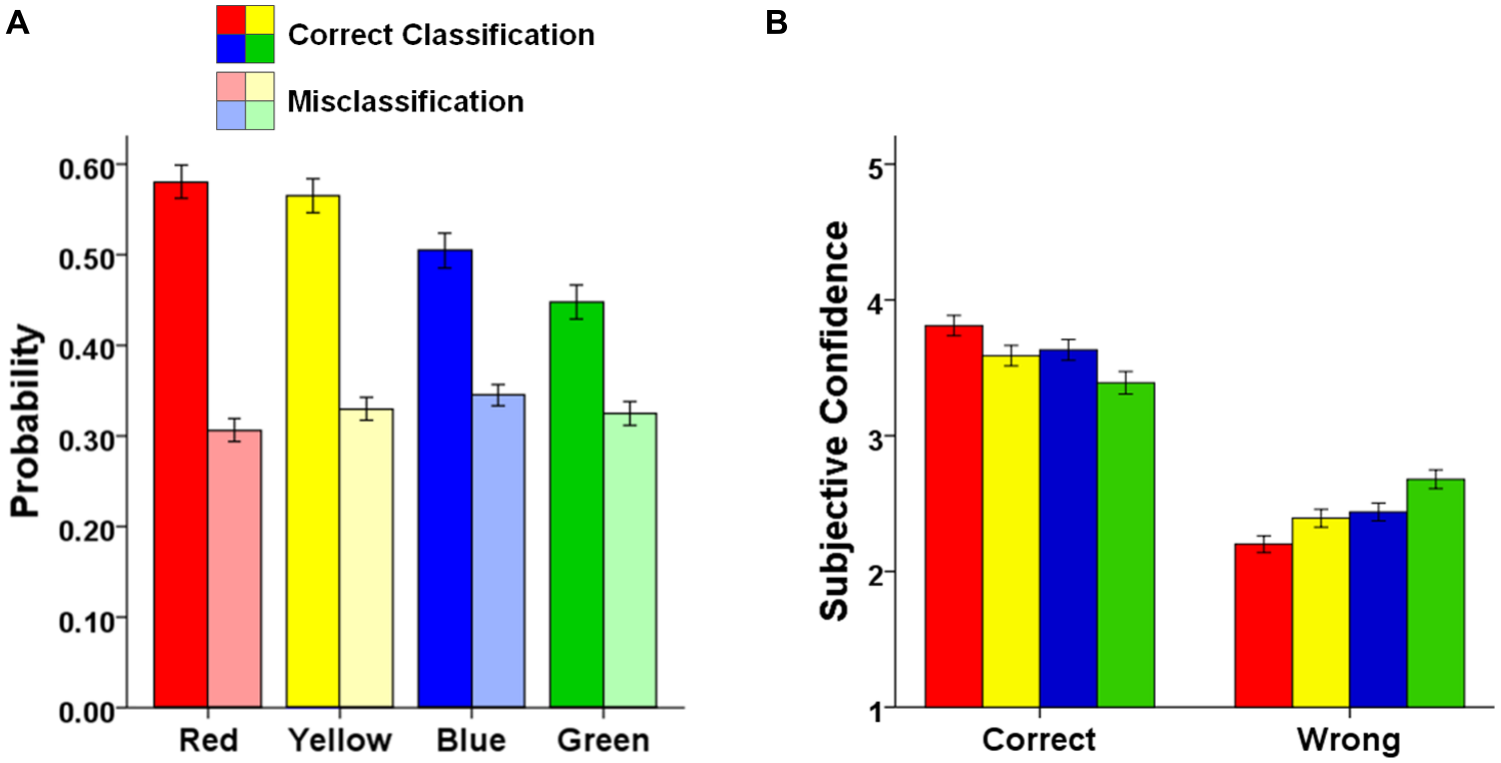
**Combined data set.** Color effects on** (A)** the probability of correctly classifying an object’s color (saturated colors) and erroneously reporting a color in case of misclassification (pale colors), and** (B)** subjective confidence in correct and wrong color memories for the combined data set. Error bars represent SE of the means.

As shown in **Figure 4A**, the probability of correctly classifying the color in which an object was presented varied as a function of color type [*F*(3,546) = 19.25, *MSE* = 0.035, *p* < 0.001, $\eta_{p}^{2}$ = 0.10]. The probability of correctly classifying the color of an object was higher for red or yellow-colored objects than for blue or green-colored objects [red vs. blue: *t*(183) = 3.94, *p* < 0.001, *d* = 0.29; red vs. green: *t*(183) = 6.86, *p* < 0.001, *d* = 0.51; yellow vs. blue: *t*(183) = 3.28, *p* = 0.001, *d* = 0.24; yellow vs. green: *t*(183) = 6.06, *p* < 0.001, *d* = 0.45], and also higher for blue than for green-colored objects [*t*(183) = 2.72, *p* = 0.007, *d* = 0.20]; color memory did not differ between red and yellow-colored objects [*t*(183) = 0.34, *p* = 0.734, *d* = 0.06]. By contrast, the probability of erroneously reporting one of the remaining color when the color of an object was misclassified did not differ between color types [*F*(3,543) = 1.22, *MSE* = 0.038, *p* = 0.301, $\eta_{p}^{2}$ = 0.007]^3^.

### SUBJECTIVE CONFIDENCE

To this point, we have demonstrated that memory for the color of an object is high for red-colored and yellow-colored objects, and particularly low for green-colored objects. To examine whether this pattern observed in objective memory performance is reflected in subjective confidences in color memories as well, we examined the participants’ confidence in their color memories for the combined data set, depending on whether the memories were actually correct or wrong (see **Figure 4B**)^4^. When an answer was correct, confidence was high for red-colored objects, medium for yellow or blue-colored objects, and low for green-colored objects [*F*(3,504) = 11.65, MSE = 0.434, *p* < 0.001, $\eta_{p}^{2}$ = 0.07]. Planned comparisons revealed that confidence ratings were significantly increased for correctly remembered red colors (all *t*s > 2.93, *p*s < 0.004), and significantly decreased for correctly remembered green colors (all *t*s < -3.18, *p*s < 0.002), compared to each of the other three colors. When an answer was wrong, the reversed pattern occurred. Confidence when making errors was low for red-colored objects, medium for yellow or blue-colored objects, and high for green-colored objects [*F*(3,492) = 17.86, MSE = 0.358, *p* < 0.001, $\eta_{p}^{2}$ = 0.10]. Confidence ratings were significantly decreased when the color of red objects was falsely remembered (all *t*s < -2.93, *p*s < 0.004), and significantly increased when the color of green objects was falsely remembered (all *t*s > 3.55, *p*s < 0.001), compared to each of the other three colors. Thus, observers were not only more likely to remember the color of an object if it was red, they were also more accurate in their confidence ratings of color memories in case of red. By contrast, in case of green colors, observers were not only more prone to making mistakes when trying to remember the color of an object, but they also were more confident in their errors.

## GENERAL DISCUSSION

Taken together, our results provide strong evidence that the binding of colors into object memory representations differs for different types of colors. When objects were colored in red or yellow, color was more strongly bound to objects in memory, compared to when objects were colored in blue or green, which was the color that was most poorly bound. Such a finding indicates that feature binding in memory is not a uniform process by which any attended feature of a stimulus is automatically bound into a unitary memory representation. Rather, our results suggest that binding in memory can vary across different subtypes of features, a finding that supports recent findings showing that the features of objects are stored in visual memory rather independently from each other (Fougnie and Alvarez, 2011; Brady et al., 2013).

The observed pattern of results is consistent with the idea that colors signal the importance of objects (Edmunds, 1974; Nunn, 1999; Dominy and Lucas, 2001). In both animals and humans, objects which are of particular significance for one’s own survival often show red colors (Parsons, 1995; Elliot and Niesta, 2008). Like red, yellow has also been linked to aposematism in insects and reptiles (Ruxton et al., 2004; Stevens and Ruxton, 2012), and is commonly used as a warning signal in human culture (Parsons, 1995). Accordingly, a stronger binding of red and yellow colors might be adaptive to retain the significance of individual objects for one’s issues and goals. Green, by contrast, is the color which is most frequent in nature due to the fact that chlorophyll, which is used by most of the plants to gain energy, does not absorb green light. Thus, green might be the color which is least informative because simply almost everything is green. Indeed, as opposed to signal colors like red or yellow, animals use green color as camouflage to blend with their environment, a behavior which is also imitated by humans by wearing green clothing in military and similar fields.

The finding that binding is stronger for red and yellow colors compared to blue and green colors is also in line with previous findings on the effects of colors on attention. As often noted in applied contexts such as advertisement and design, warm colors such as red and yellow seem to attract more attention, whereas cool colors such as blue and green seem to attract less attention (e.g., Graham, 2005). Indeed, this has been supported by more basic research, showing that warm colors are more salient than cool colors (e.g., Gelasca et al., 2005; Frey et al., 2011). For instance, using a visual search paradigm, Lindsey et al. (2010) have recently demonstrated that search times are shortest for (desaturated) warm colors, such as red and orange, and longest for (desaturated) cool colors, such as blue and green, suggesting that warm colors more strongly attract attention. Accordingly, because attention is assumed to be one of the main prerequisites for the binding of features into object representations (e.g., Treisman and Gelade, 1980), differential attentional attraction may represent the cognitive mechanism that underlies the differential binding of colors in memory. Indeed, the assumption that increased attention to a color feature can produce an enhanced binding of that feature is also supported by the memory results for oddball color pictures in Experiment 2. Gray colors that seem not to serve any signaling function (e.g., Parsons, 1995; Stevens and Ruxton, 2012) were nevertheless strongly bound into memory representations when representing oddball features that are known to attract attention due to the standing out from the context (e.g., Remington et al., 1992).

In the present experiments, participants were asked to provide a categorical memory response rather than to reproduce the exact colorimetric properties of the remembered colors. Accordingly, the question arises whether the observed differences in correctly classifying the color of differentially colored objects reflects color effects at the level of perceptual color experiences or at the level of conceptual color categories. While our data do not allow to draw conclusions on the ability to remember the exact colorimetric properties of the color of objects, the fact that a similar pattern was found even when objects were processed without any intention of memorization seems to rule out the possibility that the observed effects were based on associations between objects and conceptual color categories because it seems unlikely that color category names are activated under incidental learning conditions.

In all four experiments, stimuli of low evolutionary significance were used that were not pre-experimentally associated with any particular color. Doing so, we found that memory for the color a stimulus was particularly high for objects that were colored in red or yellow, and particularly low for objects colored in green. As mentioned above, such a pattern is well in line with evolutionary considerations, suggesting that red and yellow colors serve as signals indicating an object’s significance for one’s own survival both in animals and humans (Parsons, 1995; Elliot and Niesta, 2008; Stevens and Ruxton, 2012). Such an account would predict that the effects of color type on feature binding in memory may even be much larger for stimuli that have a higher degree of evolutionary significance (e.g., a person of the opposite sex dressed in red, blue, yellow, or green, see e.g., Elliot and Niesta, 2008, or fruits differing in ripeness, e.g., Dominy and Lucas, 2001). Thus, further exploring the role of evolutionary significance in color binding may be an important avenue for further research.

The results of the present study may be of considerable importance for basic color research and research on information processing in the field of cognitive psychology in general because numerous studies have used colors rather arbitrary to examine a variety of cognitive functions without taking into account the possibility of systematic effects of different colors. Furthermore, our results may be important in a variety of applied settings like, for instance, eyewitness testimony. An eyewitness is often asked to recall information about the color of a person’s clothes, the color of a car, or the color of other objects associated with a witnessed event. Our findings suggest that the probability of remembering color features of a critical event is not equal for different colors. Instead, eyewitness memories should be more likely to include the color of objects when they were red or yellow, whereas it should be harder to remember color features when objects were blue or especially green. In addition, the subjective confidence of eyewitnesses in their color memories should differentiate well between objectively correct and incorrect color memories in case of red-colored objects, but poorly in case of green-colored objects. In other words, if you were a smart gangster, you should drive a green rather than a red or a yellow car.

## Conflict of Interest Statement

The authors declare that the research was conducted in the absence of any commercial or financial relationships that could be construed as a potential conflict of interest.

## Footnotes

1 We use the term ‘color type’ in the sense of Berlin and Kay (1969) who defined basic color categories which are organized around universally shared focal points in color space.

2 When considering only correct color responses rated as “sure” or “very sure” as correctly remembered, color memory was high for red colors (*M* red = 41.3%), medium for yellow and blue colors (*M* yellow = 37.7%; *M* blue = 36.5%), and low for green colors (*M* green = 24.6%).

3 Due to slight differences in the procedure between the four studies (i.e., additional color gray in Experiment 2), it was not possible to apply a multinomial model to the combined data set.

4 Subjective confidence judgments were analyzed for the combined data set in order to get a more robust estimate of the effects of colors on subjective confidence ratings.

## References

[B1] Batchelder W. H. Riefer D. M. (1999). Theoretical and empirical review of multinomial process tree modeling. *Psychon. Bull. Rev.* 6 57–86 10.3758/BF03210812 12199315

[B2] Berlin B. Kay P. (1969). *Basic Color Terms: Their Universality and Evolution.* Berkeley, CA: University of California Press.

[B3] Borges M. A. Stepnowsky M. A. Holt L. H. (1977). Recall and recognition of words and pictures by adults and children. *Bull. Psychon. Soc.* 9 113–114 10.3758/BF03336946

[B4] Boynton R. M. MacLaury R. E. Uchikawa K. (1989). Centroids of color categories compared by two methods. *Color Res. Appl.* 14 6–15 10.1002/col.5080140105

[B5] Brady T. F. Konkle T. Alvarez G. A. Oliva A. (2013). Real-world objects are not represented as bound units: independent forgetting of different object details from visual memory. *J. Exp. Psychol. Gen.* 142 791–808 10.1037/a0029649 22905874

[B6] Cuvo A. J. (1975). Developmental differences in rehearsal and free recall. *J. Exp. Child Psychol.* 19 265–278 10.1016/0022-0965(75)90090-9

[B7] Dominy N. J. Lucas P. W. (2001). Ecological importance of trichromatic vision to primates. *Nature* 410 363–366 10.1038/35066567 11268211

[B8] Edmunds M. (1974). *Defence in Animals: a Survey of Anti-predator Defences*. Harlow: Longman.

[B9] Elliot A. J. Maier M. A. (2014). Color Psychology: effects of perceiving color on psychological functioning in humans. *Annu. Rev. Clin. Psychol.* 65 95–120 10.1146/annurev-psych-010213-115035 23808916

[B10] Elliot A. J. Maier M. A. Moller A. C. Friedman R. Meinhardt J. (2007). Color and psychological functioning: the effect of red on performance attainment. *J. Exp. Psychol. Gen.* 136 154–168 10.1037/0096-3445.136.1.154 17324089

[B11] Elliot A. J. Niesta D. (2008). Romantic red: red enhances men’s attraction to women. *J. Pers. Soc. Psychol.* 95 1150–1164 10.1037/0022-3514.95.5.1150 18954199

[B12] Elliot A. J. Niesta Kayser D. Greitemeyer T. Lichtenfeld S. Gramzow R. H. Maier M. A. (2010). Red, rank, and romance in women viewing men. *J. Exp. Psychol. Gen.* 139 399–417 10.1037/a0019689 20677892

[B13] Fine I. MacLeod D. I. A. Boynton G. M. (2003). Surface segmentation based on the luminance and color statistics of natural scenes. *J. Opt. Soc. Am. A Opt. Image Sci. Vis.* 20 1283–1291 10.1364/JOSAA.20.001283 12868634

[B14] Fougnie D. Alvarez G. A. (2011). Object features fail independently in working memory: evidence for a probabilistic feature-store model. *J. Vis.* 11 6 10.1167/11.12.3 PMC3279121 21980189

[B15] Fraser T. Banks A. (2004). *Designer’s Color Manual: The Complete Guide to Color Theory and Application*. San Francisco, CA: Chronicle Books.

[B16] Frey H. Wirz K. T. Willenbockel V. Betz T. Schreiber C. Troscianko T. (2011). Beyond correlation: do color features influence attention in rainforest? *Front. Hum. Neurosci.* 5:36 10.3389/fnhum.2011.00036 PMC3079176 21519395

[B17] Gelasca E. D. Tomasic D. Ebranhimi T. (2005). “Which colors best catch your eyes: a subjective study of color saliency,” in *Proceedings of First International Workshop on Video Processing and Quality Metrics for Consumer Electronics* (Washington: SPIE).

[B18] Graham L. (2005). *Basics of Design: Layout and Typography for Beginners* 2nd Edn. New York: Thomson Delmar Publishing.

[B19] Hård A. Sivik L. (1981). NCS–Natural Color System: a Swedish standard for coloer notation. *Col. Res. Appl.* 6 129–138 10.1002/col.5080060303

[B20] Heider E. R. (1972). Universals in color naming and memory. *J. Exp. Psychol.* 93 10–20 10.1037/h0032606 5013326

[B21] Hutchings J. (1997). “Colour in plants, animals, and man,” in *Color for Science, Art, and Technology* ed. Nassau K. (Amsterdam: Elsevier) 222–246.

[B22] Ioannidis J. P. A. Trikalinos T. A. (2007). An exploratory test for an excess of significant findings. *Clin. Trials* 4 245–253 10.1177/1740774507079441 17715249

[B23] Khan S. A. Levine W. J. Dobson S. D. Kralik J. D. (2011). Red signals dominance in male rhesus macaques. *Psychol. Sci.* 22 1001–1003 10.1177/0956797611415543 21750249

[B24] Kuhbandner C. Pekrun R. (2013). Joint effects of emotion and color on memory. *Emotion* 13 375–379 10.1037/a0031821 23527500

[B25] Labrecque L. I. Milne G. R. (2011). Exciting red and competent blue: the importance of color in marketing. *J. Acad. Mark. Sci.* 40 711–727 10.1007/s11747-010-0245-y

[B26] Lindsey D. T. Brown A. M. Reijnen E. Rich A. N. Kuzmova Y. I. Wolfe J. M. (2010). Color channels, not color appearance or color categories, guide visual search for desaturated color targets. *Psychol. Sci.* 21 1208–1214 10.1177/0956797610379861 20713637 PMC3050514

[B27] Mannhaupt H.-R. (1983). Produktionsnormen für verbale Reaktionen zu vierzig geläufigen Kategorien. *Sprache Kognit.* 2 264–278.

[B28] Mehta R. Zhu R. (2009). Blue or red? Exploring the effect of color on cognitive task performances. *Science* 323 1226–1229 10.1126/science.1169144 19197022

[B29] Nunn C. L. (1999). The evolution of exaggerated sexual swellings in primates and the graded-signal hypothesis. *Anim. Behav.* 58 229–246 10.1006/anbe.1999.1159 10458874

[B30] Parsons K. C. (1995). Ergonomics of the physical environment: international ergonomics standards concerning speech communication, danger signals, lighting, vibration and surface temperatures. *Appl. Ergon.* 26 281–292 10.1016/0003-6870(95)00041-A 15677031

[B31] Ragas M. C. Kozlowski K. (1998). *Read my Lips: A Cultural History of Lipstick*. San Francisco, CA: Chronicle Books.

[B32] Regier T. Kay P. Cook R. S. (2005). Focal colors are universal after all. *Proc. Natl. Acad. Sci. U.S.A.* 102 8386–8391 10.1073/pnas.0503281102 15923257 PMC1149449

[B33] Remington R. W. Johnston J. C. Yantis S. (1992). Involuntary attentional capture by abrupt onsets. *Percept. Psychophys.* 51 279–290 10.3758/BF03212254 1561053

[B34] Ruxton G. D. Sherratt T. N. Speed M. P. (2004). *Avoiding Attack: The evolutionary Ecology of Crypsis, Warning Signals, and Mimicry*. New York: Oxford University Press 10.1093/acprof:oso/9780198528609.001.0001

[B35] Schulz M. F. Sanocki T. (2003). Time course of perceptual grouping by color. *Psychol. Sci.* 14 26–30 10.1111/1467-9280.01414 12564750

[B36] Snodgrass J. G. Vanderwart M. (1980). A standardized set of 260 pictures: norms for name agreement, image agreement, familiarity, and visual complexity. *J. Exp. Psychol. Hum. Learn.* 6 174–215 10.1037//0278-7393.6.2.174 7373248

[B37] Spence I. Wong P. Rusan M. Rastegar N. (2006). How color enhances visual memory for natural scenes. *Psychol. Sci.* 17 1–6 10.1111/j.1467-9280.2005.01656.x 16371136

[B38] Stevens M. Ruxton G. D. (2012). Linking the evolution and form of warning coloration in nature. *Proc. Biol. Sci.* 279 417–426 10.1098/rspb.2011.1932 22113031 PMC3234570

[B39] Treisman A. M. Gelade G. (1980). A Feature-Integration theory of attention. *Cogn. Psychol.* 12 97–136 10.1016/0010-0285(80)90005-5 7351125

[B40] Treisman A. Schmidt H. (1982). Illusory conjunctions in the perception of objects. *Cogn. Psychol.* 14 107–141 10.1016/0010-0285(82)90006-8 7053925

[B41] Uchikawa K. Shinoda H. (1996). Influence of basic color categories on color memory discrimination. *Color Res. Appl.* 21 430–439 10.1002/(SICI)1520-6378(199612)21:6<430::AID-COL5>3.0.CO;2-X

[B42] Wheeler M. E. Treisman A. M. (2002). Binding in short-term visual memory. *J. Exp. Psychol. Gen.* 131 48–64 10.1037//0096-3445.131.1.48 11900102

[B43] Wichmann F. A. Sharpe L. T. Gegenfurtner K. R. (2002). The contributions of color to recognition memory for natural scenes. *J. Exp. Psychol. Learn. Mem. Cogn.* 28 509–520 10.1037//0278-7393.28.3.509 12018503

[B44] Wyszecki G. Stiles W. S. (1982). *Color Science.* New York: Wiley.

